# The Impact of Wildfires on Mental Health: A Scoping Review

**DOI:** 10.3390/bs11090126

**Published:** 2021-09-21

**Authors:** Patricia To, Ejemai Eboreime, Vincent I. O. Agyapong

**Affiliations:** Department of Psychiatry, Faculty of Medicine and Dentistry, University of Alberta, Edmonton, AB T6G 2B7, Canada; eboreime@ualberta.ca (E.E.); agyapong@ualberta.ca (V.I.O.A.)

**Keywords:** wildfire, bushfire, mental health, major depressive disorder, anxiety disorder, post-traumatic stress disorder, substance use, resilience

## Abstract

One of the many consequences of climate change is an increase in the frequency, severity, and, thus, impact of wildfires across the globe. The destruction and loss of one’s home, belongings, and surrounding community, and the threat to personal safety and the safety of loved ones can have significant consequences on survivors’ mental health, which persist for years after. The objective of this scoping review was to identify primary studies examining the impact of wildfires on mental health and to summarize findings for PTSD, depression, anxiety, and substance use. Literature searches on Pubmed and Embase were conducted in February and April of 2021, respectively, with no date restrictions. A total of 254 studies were found in the two database searches, with 60 studies meeting the inclusion criteria. Three other studies were identified and included based on relevant in-text citations during data abstraction. The results show an increased rate of PTSD, depression, and generalized anxiety at several times of follow-up post-wildfire, from the subacute phase, to years after. An increased rate of mental health disorders post-wildfire has been found in both the adult and pediatric population, with a number of associated risk factors, the most significant being characteristics of the wildfire trauma itself. Several new terms have arisen in the literature secondary to an increased awareness and understanding of the impact of natural disasters on mental health, including ecological grief, solastalgia, and eco-anxiety. There are a number of patient factors and systemic changes that have been identified post-wildfire that can contribute to resilience and recovery.

## 1. Introduction

Warmer temperatures, longer summers, and blistering heat waves are all consequences of climate change and contributing factors to an increasing awareness and incidence of wildfires [1]. The three key ingredients necessary to start a fire are heat, oxygen, and fuel [2]. Consequences of climate change include warmer temperatures and changes in wind speed and rainfall patterns, the very factors necessary for wildfire ignition and propagation [3]. The World Health Organization (WHO) estimates that wildfires and volcanic activities impacted 6.2 million people globally from 1998 to 2017 [4]. Current statistics reveal an ongoing significant impact of wildfires across the globe. For example, in 2020, the United States reported that by October 2020 8 million acres of land had been burned by wildfires [5]. On average, Canada has spent 800 million dollars annually on wildfire related costs in recent years [6]. Similarly, Australia has reported an increase of 30.6 days in the annual number of average days with high-extreme fire danger from 2016 to 2019 [7]. Additionally, contributing to the greater human experience of and impact from wildfires are changes in human migration and expansion. The pattern of human expansion into areas previously dominated by wildlife and nature means more people now live close to, or in, wooded areas, which serve as the fuel and setting for wildfires [8]. Researchers calculate that global wildfires can produce between 1.75 and 13.5 billion metric tons of carbon annually which circulate in our atmosphere for months [9]. Thus, wildfires themselves contribute to the greenhouse effect, propagating further development of wildfires.

Wildfires exert various impacts and risks to public health including an acute risk of mortality and injury, and by wreaking havoc on the community and the ensuing response team. Wildfires destroy homes and workplaces, displace victims, and impact numerous medical conditions such as chronic obstructive pulmonary disease (COPD), asthma, and mental health [10,11,12]. Studies have found higher rates post-wildfire of hypertension, gastrointestinal disorders, diabetes, and COPD and asthma exacerbation, along with various psychiatric conditions, including mood and anxiety disorders [11,12,13,14,15]. The impact of wildfires on the survivors’ mental health has been found in both the adult and pediatric population, with children and adolescents also experiencing higher rates of mood and anxiety disorders post-wildfire [16,17]. Researchers studying the impact of wildfires on children and adolescents have utilized survey and scale measures administered to study participants in this demographic, as well as their parents and/or teachers, to gain an appreciation of mental health outcomes within a study population that may struggle with personally communicating mood symptoms secondary to their developmental level [16,18,19]. Behavioral changes post-wildfire in children can include increased irritability and changes in concentration, sleep, and academic performance [18,20]. The impact of wildfires on mental health cannot be ignored and should not be, as the World Health Organization (WHO) identified neuropsychiatric disorders as the leading cause of disability-adjusted life years in the U.S. in 2010 with mental and behavioural disorders contributing to 13.6% of the total [21]. The repercussions from wildfires can be widespread due to the ability of wildfire-created smoke to dissipate and persist, with the majority of follow-up studies being conducted within 1 to 2 years post-wildfire. However, there is evidence of ongoing mental health effects in more longitudinal studies made 10–20 years post-wildfire [22,23]. There are novel terms to describe emotional and mental health responses to natural disasters such as solastalgia, eco-anxiety, and ecological grief, which will likely become more prominent as such tragedies continue to occur [24,25,26].

This paper is a scoping review of the existing literature, investigating the research question of how wildfires impact mental health; specifically, post-traumatic stress disorder (PTSD), major depressive disorder/depression (MDD), generalized anxiety disorder (GAD), and substance use at various times of follow-up. Our objective was to summarize the existing information on the impact of wildfires on mental health, and contribute to the understanding of factors that contribute to and protect against psychopathology post-wildfire. This information may be utilized to determine evidence-based public health responses to mitigate adverse outcomes. To our knowledge, this is the first scoping review to specifically examine the mental health effects of wildfires.

Through our review, we hope to summarize specifically and comprehensively how wildfires impact mental health and discuss how this information may be utilized for future research, wildfire disaster response, and ultimately improving population health.

## 2. Materials and Methods

A literature search on 16 February 2021, was first conducted in Pubmed, with no date restrictions or language filters. Search terms included “post traumatic stress disorder”) OR PTSD OR psychosis OR schizophreni* OR depressi* OR anxiety OR “mental illness*” OR “psychiatric disorder*” OR “psychiatric illness*” OR “mental disorder*” OR “mental health” AND wildfire* or forestfire* or bushfire* OR wild n6 fire* OR bush n6 fire* OR forest n6 fire* OR vegetation n6 fire* OR landscape n6 fire*.

Then a literature search on 7 April 2021, was conducted on Embase/OVID in their 1974 to present database, with no language filters. Search terms included exp mental disease/, exp wildfire/, (post traumatic stress disorder or PTSD or psychosis or schizophreni* or depressi* or anxiety or mental illness* or psychiatric disorder* or psychiatric illness* or mental disorder* or mental health).mp., (wildfire* or forestfire* or bushfire* or brushfire*).mp., and ((wild or brush or bush or forest or vegetation or landscape or peat or peatland) adj3 fire*).mp. The final search included all these terms together. 

### 2.1. Inclusion Criteria

This review contains primary articles and includes randomized controlled trials and observational studies, such as case-control or cross-sectional studies, which reported on PTSD, anxiety, depression, substance use, or resiliency measures as outcomes of interest following wildfire exposure. Participants in the studies came from a wide breadth of age categories, from primary school children to elderly adults in their 90′s. 

### 2.2. Exclusion Criteria

1. Reviews and subjective reports without any new objective primary data were excluded from this review.
2. Studies focusing primarily on firefighters, emergency responders, or burn victims were excluded.
3. Studies were excluded if the exposure was non-wildfire based or the primary outcome was not related to mental health.
4. Studies with a primary outcome of schizophrenia and psychosis were excluded due to the limited number of total studies available.

### 2.3. Data Extraction

The primary author reviewed the articles to determine which studies met the inclusion criteria and then performed the data extraction based on outcome measures of interest. 

## 3. Results

Our review appraised and synthesized evidence from the literature about the impact of wildfires on mental health, specifically with respect to PTSD, depression, anxiety, and substance use. We present the findings of our search strategy and then findings on our primary aim under the various clinical outcomes.

### Outcome of Literature Search

**Figure 1:**
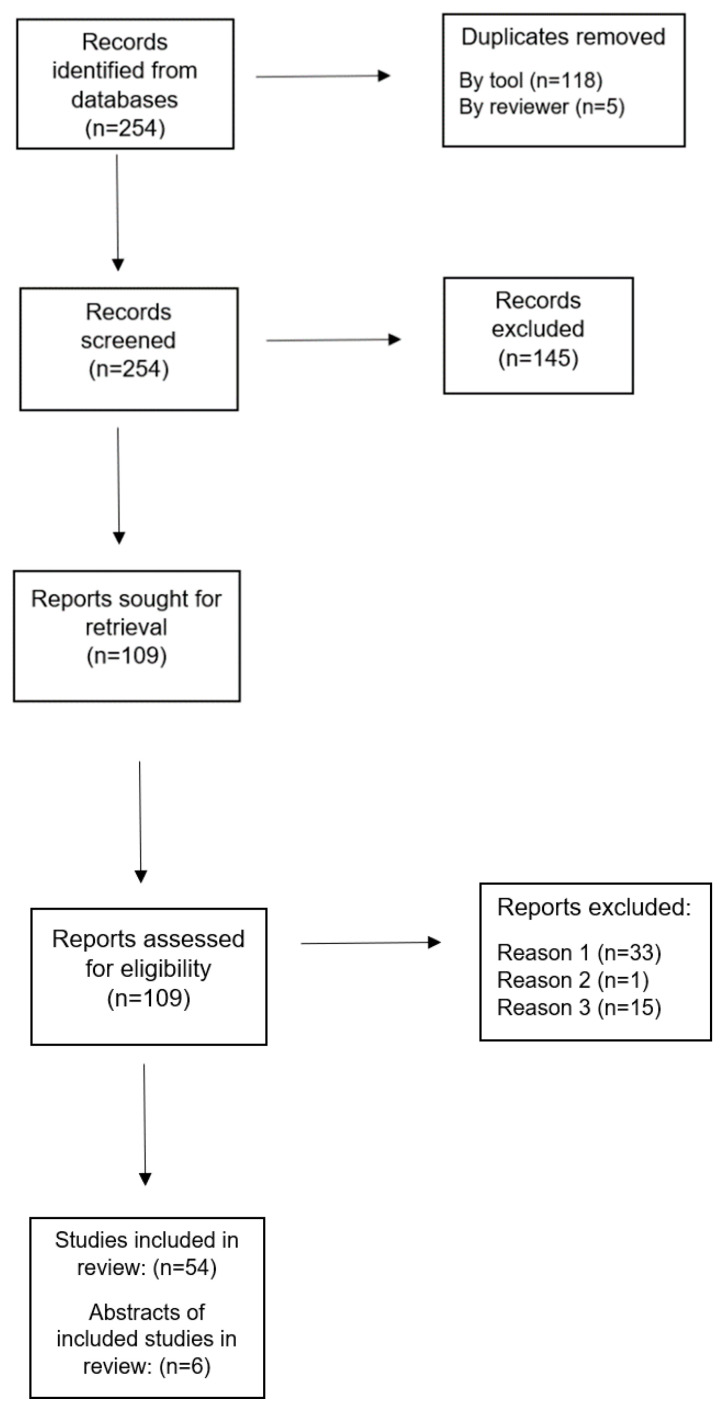
Literature Search Flow Chart.

Figure 1 is a flowchart summarizing our search process and outcomes. This review found 60 articles that met the inclusion criteria [13,14,16,17,19,20,22,23,26,27,28,29,30,31,32,33,34,35,36,37,38,39,40,41,42,43,44,45,46,47,48,49,50,51,52,53,54,55,56,57,58,59,60,61,62,63,64,65,66,67,68,69,70,71,72,73,74,75,76,77]. Three other articles were obtained from review of relevant in-text citations during data abstraction [18,78,79].

Table 1 details study characteristics. The earliest study was published in 1985 by Clayer et al. [13]. The vast majority (n = 57, 90%) were published from the year 2000 and onward. The results of the included studies are summarized in Table 2.

## 4. Discussion

This scoping review is specific to the impact of wildfires on mental health in both the adult and pediatric population. There are a number of narrative reports and letters discussing the impact of wildfire by experts in the field [5,80]. Our literature search identified one review specific to the impact of wildfire smoke exposure, which included a number of health outcomes including mental health [81]. In addition, there are several reviews that discussed the impact of wildfires on mental health within the broader context of natural disasters or climate change [12,25,82].

### 4.1. Post-Traumatic Stress Disorder

A post-natural disaster acute stress reaction is common and expected, but the rate at which an individual’s acute stress reaction may persist and develop into psychopathology is a common research objective [12]. Researchers have attempted to quantify the rates and severity of PTSD at various times post-wildfire in children, adolescents, and adults. This review suggests there are statistically and clinically significant increases in rates of PTSD in communities ravaged by wildfires. Among adults, higher rates of PTSD and associated symptoms were present shortly after a wildfire and up to 10 years post-wildfire [22,36]. In the adult population, the rate of probable PTSD based on a survey screening 3 months post-wildfire was found to be 24% and 60% in two separate studies [36,57]. At 6 months after a wildfire, the one month prevalence rate of PTSD in adults was 12.8%–26% [34,75]. At 18 months after the Fort McMurray wildfire, rates of likely PTSD continued to be higher than prior to the trauma, with studies finding similar rates, within 10.2%–13.6% [32,64,71]. Bryant et al. provided unique longitudinal information about the impact and prevalence of psychopathology post-wildfire [22,26,42]. From 3 to 10 years post-wildfire, the rate of fire-related PTSD in the high-impact group decreased from 15.6% to 7.6% [22,26,42]. 

Studies have looked at other outcomes or symptoms associated with PTSD post-wildfire including sleep disturbance and insomnia, anger, attachment style, interpersonal violence, and a term known as vicarious traumatization which is the development of PTSD symptoms from indirect trauma exposure [43,46,48,50,51,62,70,73].

A unique and recurring wildfire experience in South Asian countries is the seasonal haze created by bushfires from the intentional slash-and-burn technique used for clearing land for agricultural purposes [55]. Respondents who perceived lower Pollutants Standard Index values as dangerous were associated with higher IES-R scores [55]. In comparison, a study in British Columbia, Canada, on the impact of increased particulate matter (PM) and worsening air quality related to wildfires did not find an association with an increase in mental health related physician visits [63]. 

Rates of psychopathology post-wildfire exposure have also been studied in the pediatric population. In the sub-acute phase post-wildfire, the number of children with significant PTSD symptoms can be as high as 92% [16]. Studies that have assessed the rate of likely PTSD 6 months post-fire have found similar results with 9–12% of children and adolescents experiencing moderate to severe PTSD and as high as 29.4% in a study involving adolescents in Greece [19,58,65]. A year post-wildfire, the rate of PTSD in children and adolescents has been found to be between 27 and 37% [17,76]. The impact of a childhood exposure to a bushfire on one’s mental health as an adult was evaluated with a 20 year follow-up study conducted by Mcfarlane and Van Hooff [23]. They found no difference in the rate of lifetime or recent 1-month prevalence of PTSD in the wildfire exposed group versus controls [23]. 

Our literature search did not reveal a review specific to risk factors for developing PTSD post-bushfire, but such factors have been investigated by several primary studies. In adults, demographic factors such as female gender, widowed status, or fewer years of education, lower socioeconomic status, and non-caucasian ethnicity are associated risk factors, although gender was not always been found to be significant [26,32,69,73,78]. Trauma-related factors significant to increasing one’s risk of post-wildfire PTSD include personal witnessing of burning homes, having fear for one’s life or lives of loved ones, losing a loved one, significant property damage, or feeling a lack of support from family, friends, and/or the government [22,26,32,34,42,57,62,69]. The trauma related factors, including perceived threat to one’s life and subsequent reaction to the trauma, were found to be more contributory to the degree of PTSD symptoms than demographic or pre-trauma factors [69]. Finally, a contributing factor to the risk of developing PTSD post-bushfire is ongoing trauma and life stressors [22,23,26,42]. The risk factors for PTSD in children and adolescents were overall quite similar to adults, including demographic factors like female gender, younger age, and middle grades 7–9 [19,58,59,65,76]. For children, a greater predictor of increased emotional distress post-wildfire was the fear for their parents’ lives, even more than fear for their own life [19]. Combined with the identified risk factor in adults of a perceived lack of support from loved ones or the government, there is significant evidence to support the critical role a government and public health response has in mitigating mental health consequences after experiencing a bushfire [71]. A tangible government response may serve as validation that a traumatic experience has occurred, whilst providing avenues for recovery and moving forward. The impact and contribution ongoing adverse life events have on the risk of developing psychopathology post-wildfire is indicative of how cumulative stressors may deteriorate human resilience. As such, government aid should not be time sensitive post-wildfire, but rather be sustained in order to mitigate ongoing life stressors that can occur after a community is disrupted.

### 4.2. Depression

Second to PTSD, major depressive disorder (MDD) is one of the most common psychiatric illnesses studied and screened-for post-natural disasters [83]. Similar to rates of PTSD, there has also been shown to be higher rates of depression and associated symptoms post-wildfire in adults, which can persist up to 10 years [22,57]. In the adult population, studies have found rates of depression approximately 3 months post-bushfire, between 25.5 and 33% [36,57]. At 6 months post-wildfire, the estimated rates of MDD are between 10.4% and 17.1%, and between 18.3 and 24.8% at 18 months post-wildfire [32,33,64,71]. Finally, Bryant et al. studied a range of psychological outcomes over 10 years following the devastation of the Victorian Black Saturday bushfire including depression [22,26,42]. The rate of depression in the high-impact group was consistently around 10% at the three periods of follow-up [22,26,42].

Rates of depression post-wildfire have also been studied in children and adolescent populations. Mcdermott et al. were some of the first researchers to look at depression in children 6 months post-fire and found a rate of 4.7% [19]. However, a more recent study found a higher rate of depression 6 months post-wildfire at 20% [65]. Even at 18 months post-wildfire, one third of grade 7–12 students were found to meet criteria for depression compared to 17% in an age-matched control group [17,39]. 

The emotional response of solastalgia, which describes the mourning of changes in one’s natural environment, has been linked to levels of psychological distress post-wildfire [84]. Approximately one year after a wildfire, 35% of the participants had scores indicative of moderate-high risk for depression or anxiety on the Kessler Psychological Distress scale, and increased solastalgia was associated with greater odds of psychological distress [84].

There were fewer studies in the literature search investigating risk factors for depression post-bushfire in comparison to PTSD. However, some patient factors that have been identified in adults include female gender, age greater than 40 years old, a greater number of adverse experiences in childhood, a prior history of depressive disorder or anxiety disorder, and prior treatment with an antidepressant [14,33,64,71,73]. Other researchers did not find an association between demographic factors or gender and depression [57,78]. Trauma factors that increase risk of depression post-fire in adults are similar to PTSD: witnessing or experiencing property loss and fear for safety of loved ones [22,26,32,57,64]. Identified post-trauma risk factors include perceived lack of support from family, friends, or the government, ongoing life stressors, and having received counselling post-fire [22,26,32,33,42,64]. Similar risk factors for depression post-wildfire have also been identified in the pediatric population; although female gender has not been a consistent statistically significant risk factor, the trend is for higher symptom scores [58,59,65]. Finally, studies have commented on the association between PTSD and depression in children and adults, with a co-occurrence rate of 0.74 in adults [19,57]. 

Future research may utilize screening measures that combine specific depression related questions with associated behavioral changes that impact children’s functioning at home and school. Involving not only children and adolescent participants, but their parents, caregivers, and teachers in assessments would likely result in a more comprehensive screening for psychopathology post-wildfire.

### 4.3. Anxiety

There have been a number of studies investigating rates of anxiety disorders post-wildfire, although the quantity of evidence is smaller than that for PTSD and depression. Studies performed post-wildfire have shown higher rates of anxiety in both adult and the pediatric populations [17,19,31,36]. In adults surveyed 3 months post-wildfire, 27.0% met criteria for an anxiety disorder other than panic disorder and 17.4% had symptoms significant for panic disorder [36]. The one-month prevalence rate for generalized anxiety disorder symptoms 6 months post-wildfire was 19.8% [31]. At 18 months post-wildfire, the authors found similar rates of generalized anxiety disorder, between 15.7 and 18.7%, with self-reported pre-wildfire rates of anxiety estimated at 8.6–14.5% [32,64,71]. Finally, Bryant et al. followed a population of adults for up to 10 years after the Victorian Black Saturday bushfire, and over that time the rate of severe distress, as measured on the Kessler Psychological Distress Scale, decreased from 7.5% to 4.4% [22,26,42]. 

Anxiety in children and adolescents’ post-wildfire has also been studied, although to a lesser extent than adults. At 6 months post-wildfire, 14.1% of children had symptoms significant for high anxiety [19]. Looking later on, at 18 months after the Fort McMurray wildfire, 27% of grade 7–12 students had suspected anxiety, with another study finding no difference in the rate of anxiety disorders between wildfire exposed and control groups [17,39]. One unique study looked at childhood exposure to the Ash Wednesday wildfires of 1983 and followed up with participants 20 years later in adulthood [23]. Only the lifetime rate of any anxiety disorder other than PTSD was found to be significant, specifically with regards to environmental phobia [23]. 

A limitation in studies exploring anxiety is the reliance on scales to objectively measure anxiety such as the Global Health Questionnaire or the Kessler Psychological Distress Scale [22,60]. This may limit the ability to make specific mental health diagnoses, such as generalized anxiety disorder, without understanding the key criterion and degree of functional impact within each participant. One mechanism by which wildfires increase victims’ anxiety is the resulting emotion and mental turmoil of uncertainty after experiencing such devastating losses and damage to oneself, loved ones, property, belongings, and livelihood. The sensation of anxiety is not necessarily maladaptive, rather it may serve a functional purpose of helping create emotional motivation to respond and prepare for a perceived future threat [85,86]. Therefore, researchers and clinicians must distinguish between anxiety that serves as a normal and healthy response to a life stressor versus a debilitating anxiety disorder. Anxiety post-wildfire may help provide fuel for appropriate reactions, such as pursuing financial support, rebuilding one’s home, identifying available community resources, and cumulatively helping a victim heal from such trauma. However, functionally impairing anxiety describes an emotional response that no longer serves the individual, and therefore the distinction between the two is critical. 

Similarly to the risk factors identified for PTSD and depression post-wildfire, the risk factors for anxiety can also be separated into patient factors, trauma factors, and post-trauma factors [31]. Patient factors include younger age or age ≤25 years old, lower socioeconomic status, a history of anxiety or depressive disorder, being on an antidepressant before the fire or prior adverse experiences in childhood [31,32,64,71,73]. Trauma related factors increasing the risk of anxiety include witnessing or experiencing property loss and experiencing or fearing the loss of loved ones [14,26,31,32,42,64]. Finally, factors that occur post-fire that may increase the risk of developing anxiety were similar to depression, including perceived lack of support and ongoing life stressors [14,26,31,32,42].

### 4.4. Substance Use

There is a paucity of evidence specific to substance use rates and disorders post-wildfire. In the resulting studies identified from the search terms, substance use post-wildfire was not explored as a primary independent outcome measure but always in combination with other psychological disorders [26,31,32,68]. It is estimated that at least 50% of individuals with a psychiatric illness will also have a comorbid substance use disorder at some point in their lives, and this number is significantly higher in certain populations or settings such as in-patient or treatment programs [87,88,89,90].

The existing studies indicate that there is an association between increased alcohol or drug use post-fire and psychiatric disorders [22,26,32,34,42,64]. For example, in adults 6 months post-wildfire, those with likely generalized anxiety disorder were three-times more likely to present with a drug related problem [31]. Studies have found the rate of alcohol use disorder or heavy alcohol use post-wildfire was 17% at 3 months post, 22% at 3 years post, and 18.6% at 10 years post, within the highly impacted community [22,36]. One study that evaluated substance use in college students 18 months after the Fort McMurray wildfire found a rate of 15.5% for high risk drinking, 13% for problematic drug use, and 4.4% for moderate-high nicotine dependence, with some gender differences in each category [71]. Some studies have found an association between drug related problems post-wildfire and mental illness but have not found the same relationship with high-risk drinking [32,34,64]. Other studies have found no difference in rates of substance or alcohol use in adults and adolescents exposed to a wildfire [14,23,39]. Thus, the exact relationship between mental illness and substance use and misuse is still unknown, but is likely reciprocal in nature due to a combination of genetic and environmental risk factors mutual to both; in addition, each disease may contribute to the development and persistence of the other through various factors such as the utilization of a substance as a coping mechanism for symptoms and the impact of substances on mood [87].

### 4.5. Resilience

Resilience is a common term found in the primary studies of this review as they investigated mental health outcomes such as PTSD, MDD, and GAD. It is a difficult concept to quantify objectively, as it represents such a complex sum of dynamic parts. Broadly, and within a psychological context, it refers to experiencing stressors and consequently negative emotional responses but adapting to such adversity with an absence of psychiatric conditions that by definition are functionally impairing [91,92]. Researchers have attempted to quantify resilience post-wildfire in patients or participants as the opposite of measures of psychopathology, i.e., low scores on psychological symptom scales or based on specific resiliency scales [22,26,42]. Although survey scales may be the best tool researchers have to quantify resiliency, they underestimate the fluidity and complexity of its constantly adapting components including patient characteristics and family and community supports. Individuals with high resilience scores after experiencing a wildfire had lower scores on screening measures for mental health disorders including PTSD, depression, and anxiety, and increased self-esteem and quality of life scores [17,73]. There is existing research on the relationship between resiliency factors such as coping strategies, group involvement, and psychotherapy with mental health outcomes post-wildfire [35,47,49,54]. Another focus of future research may be on appropriate tools and interventions from a broader public health perspective, to optimize patient resilience post-wildfire and prevent the development of psychopathology.

### 4.6. Environmentally Related Distress

Destructive natural disasters such as wildfires damage man-made structures but also the surrounding natural environment. The ensuing mourning and emotional response from the loss of nature has been termed ecological grief [24,93]. Closely related to ecological grief, and even called a sub-concept, is the term solastalgia; which is described as the feeling of homesickness whilst still at home [24]. The Aboriginal population residing in the Northwest Territories have been studied qualitatively in regards to their response to experiencing a wildfire, and a common theme was mourning their natural home and way of life [94]. The emotions linked to solastalgia also include anxiety, as it describes an emotional response that can include worry about potential environmental changes [24]. Eco-anxiety is worry or distress regarding the negative consequences of environmental changes [24,85,95,96]. The terms ecological grief, solastalgia, and eco-anxiety embody the psychological distress and reactions that can occur after a natural disaster or from climate change, and have started to appear in the literature on how wildfires impact mental health. Such terms reflect the role by which coping strategies that utilize the connection to one’s surrounding ecosystem may mitigate feelings of loss, mourning, and stress [97,98]. Qualitative evidence indicates that individuals may find a benefit in personal recovery through interactions and participation in environmental recovery [98]. This may reflect the protective factor a strong environmental connection provides by motivating the individual to participate in rebuilding processes that help their personal recovery.

### 4.7. Future Research

The existing studies show that wildfires do increase the rates of both medical and mental illness post-exposure; therefore, another area for future research may be a focus on the connection between the two, as well as the impact on less-studied outcomes such as psychosis [13]. McFarlane and Van Hooff identified limitations within the current disaster research, describing how the majority of studies are focused on follow-up within 1–2 years rather than greater longitudinal monitoring [99]. More long-term follow-up can provide key information on risk factors for persistence of psychiatric complications and information for interventions. Finally, with social media the dissemination of information is more rapid and uncontrollable than ever before. The public perception of facts and news is as important as the facts themselves, and future research could be into effective post-disaster communication, dissemination of information, and the impact on the individual. A novel method of population mental health support, which has been explored most recently throughout the COVID-19 pandemic, has been the utilization of supportive text messages [100]. Studies have found a significant reduction in anxiety, depression, and stress symptoms based on survey scores, as well as in prior randomized controlled clinical trials [100,101,102,103,104,105,106]. High user satisfaction has also been reported with supportive text message interventions [107,108,109,110]. From our literature search, text messaging as an intervention and public health response has not yet been explored in a wildfire impacted population and could serve as a novel area for research.

### 4.8. Limitations of the Study

Several of the limitations of this review were due to the constraints of time. Pubmed and Embase/OVID were chosen as the databases for the literature search, due to their accessibility and the availability of primary studies. However, including more databases in our literature search would add to the breadth of results and available data. Another limitation was that substance use was not one of our core search terms, and it was not until after the database search that we realized the volume of evidence available on rates of substance use post-wildfire and decided to include information from the included studies in this review. Other limitations included a single author performing the data extraction, an additional author would have added to the consistency of result data extracted. Moreover, not all included articles were peer reviewed, which may influence the quality of these studies. 

## 5. Conclusions

With populations continuing to live closer to nature or once forested areas, wildfires will continue to exist and pose a threat to individual wellbeing and public health. In understanding the consequences of wildfires on both medical conditions and mental disorders, there is a need for a public health response that is comprehensive, sustained, and as adaptive as the people it serves. There is a strong body of literature to support the impact of wildfires on mental health, but more information is needed on the effective public health measures and rebuilding strategies that can optimize patient resilience post-wildfire. 

## Figures and Tables

**Table 1:** Characteristics of articles included in the study.

Variable	N = 63
Country of origin of study	
Australia	26
Canada	14
United States	11
Greece	8
Israel	2
Singapore	1
Spain	1
Type of study	
Cross-sectional	42
Cohort	20
Randomized Controlled Trial	1
Outcomes studied	
Post-Traumatic Stress Disorder (PTSD)	11
Generalized Anxiety Disorder (GAD)	1
Major Depressive Disorder (MDD)	1
Combination of PTSD and/or MDD and/or GAD	30
Various other Combinations of Mental Health Outcomes/Measures	20
Number of studies including common outcome measures	
PHQ-9: Patient Health Questionnaire-9	18
PCL-5: PTSD Checklist for Diagnostic Statistical Manual V	17
AUDIT or AUDIT-C: Alcohol Use Disorder Identification Test (Consumption)	10
IES: Impact of Events Scale +/− Revised	10
SCL-90-R: Symptom Checklist 90-Revised Instrument	6
K6/10: Kessler Psychological Distress Scale	6
GAD-7: Generalized Anxiety Disorder-7	5
DUDIT: Drug Use Disorder Identification Test	4
HADS: Hospital Anxiety and Depression Scale	3

**Table 2:** Results Table.

References (Include Author and Citation #)	Country of Study	Study Aims	Study Design	Diagnoses/Outcome Measures	Main Findings
Adamis et al. [27] Abstract 2012	Greece	To assess after a wildfire how psychopathology may evolve longitudinally, 3 years after.	Comparative Cross-sectional Study	The measured variable was psychological distress, using the SCL-90-R.	Psychological distress was significantly lower 3 years post-wildfire compared to 6 months post, but victims of the disaster continued to have higher scores on the SCL-90-R.
Adamis et al. [28] Abstract 2012	Greece	To assess quality of life of adults living in a rural area impacted by a wildfire, 3 years prior.	Cross-sectional Study	The measured variables were quality of life, as assessed on the WHOQOL-BREF.	Multivariable analysis indicated that victims had a statistically significant poorer quality of life, with respect to the environmental health domain.
Adamis et al. [29] Abstract 2011	Greece	To examine risk factors and psychiatric outcomes.	Comparative Cross-sectional Study	The measured variable was psychological distress, using the SCL-90-R.	Victims of the wildfire had significantly higher scores for symptoms, had a greater number of symptoms (PST), and were more distressed by them (GSI).
Afifi et al. [30] 2012	United States	To analyze the impact of uncertainty secondary to experiencing natural disasters on mental health, and to investigate the role of communal coping	Cross-sectional Study	Measures included the MHI-5, rating personal feelings of uncertainty, and a measure for communal coping.	Residents who were evacuated experienced greater uncertainty regarding their home. In those evacuated, communal coping had a moderating role for uncertainty.
Agyapong et al. [31] 2018	Canada	To examine the prevalence rate and risk factors for GAD 6 months after a wildfire.	Cross-Sectional Study	Outcome measures included the GAD-7, the AUDIT, the DUDIT, and the Fagerstrom Test for Nicotine Dependence.	The one month prevalence rate for GAD was 19.8%, and between 7 and 14% had scores indicative of a substance use disorder.
Agyapong et al. [32] 2020	Canada	To evaluate prevalence rates for likely PTSD, MDD, and GAD 18 months after a wildfire.	Cross-sectional Study	Outcome measures included the PHQ-9, GAD-7, PCL-5, the AUDIT, and the DUDIT.	The 1 month prevalence rates for likely MDD, GAD, and PTSD were 18.3, 15.7, and 10.2%, respectively. While, 11.2% of participants reported symptoms of high-risk drinking and/or problematic drug use.
Agyapong et al. [33] Oral Communications- Abstract 2018	Canada	To evaluate the prevalence rate and risk factors for MDD 6 months after a wildfire.	Cross-Sectional Study	The PHQ-9 was used to evaluate for MDD.	The one month prevalence rate for likely MDD was 14.8% (10.4% for males, and 17.1% for females).
Agyapong et al. [34] 2019	Canada	To evaluate the prevalence rate and risk factors for PTSD 6 months after a wildfire.	Cross-sectional Study	Outcome measures included part 3 of the PCL-5, AUDIT, DUDIT, and Fagerstrom test for nicotine dependence.	12.8% of respondents (14.9% of females and 8.7% of males) met criteria for PTSD. Those with PTSD reported increased drug use.
Belleville et al. [35] Abstract 2019	Canada	To study a tool for supporting resilience and improving sleep after a wildfire.	Randomized Controlled Trial	Measures for symptoms of post-traumatic stress, depression, and insomnia.	Analyses revealed a significant improvement in post-traumatic stress, depression, and insomnia symptoms in the treatment group.
Belleville et al. [36] 2019	Canada	To evaluate psychological symptoms 3 months after a wildfire.	Cross-Sectional Study	Outcome measures included the PCL-5, the PHQ-9, Insomnia Severity Index, PSQI-A, PTCI, and WCQ.	Approximately 60% of participants had post-traumatic stress, 33.1% MDD, 27.0% for anxiety disorders other than panic, 17.4% for panic disorder, and 17.1% for alcohol abuse disorder.
Binet et al. [37] 2021	Canada	To determine if mental health service utilization post-wildfire differed by gender.	Cross-sectional Study	Outcome measures included the Perceived Need for Care Questionnaire, PCL-5, PHQ-9, and the Insomnia Severity Index.	From survey measures, 50.2% had a probable diagnosis of PTSD, 58.5% a probable diagnosis of MDD, and 57.4% a probable diagnosis of insomnia. Female gender was a statistically significant predictor of receiving mental health services.
Block et al. [38] 2019	Australia	To evaluate how the natural environment can be part of personal recovery after a wildfire.	Cross-Sectional Study	Outcome measures included self report of environmental attachment, the K6, the PCL, the PHQ-9, the CD-RISC, and the short-form for post-traumatic growth inventory.	Those with a weak attachment to the environment had higher scores of psychological distress, fire-related PTSD, and MDD symptoms, and lower levels of resiliency.
Brown et al. [17] 2019	Canada	To investigate how youth mental health is impacted 18 months after a wildfire.	Cross-Sectional Study	Outcome measures included the CPSS, PQH-A, the HADS, the CRAFFT, Tobacco Use Questionnaire, the RSE, the Kidscreen Questionnaire, and the Child and Youth Resilience Measure.	A total of 37% of the students met criteria for probable PTSD, 31% for probable depression, and 27% for probable anxiety. Students classified as high resilience had lower scores on all the mental health disorder screens and higher self-esteem and quality of life scores.
Brown et al. [39] 2019	Canada	To assess the impact of a wildfire disaster on adolescent mental health.	Comparative Cross-Sectional Study	Outcome measures included the PHQ-A, the HADS, the CRAFFT Questionnaire, the Tobacco Use Questionnaire, the RSE, the Kidscreen Questionnaire, and the CPSS.	In the wildfire exposure group, adolescents had higher depression and anxiety scores and rates of probable depression. While, 37% of the students met criteria for probable PTSD.
Bryant et al. [22] 2020	Australia	To examine the long-term psychological outcomes 10 years after a wildfire.	Cohort Study	Outcome measures included the PCL-5, PHQ-9, K6, and the AUDIT-C.	In the high-impact group, the rate of probable PTSD was 16.8% and MDE was 9.8%. The likelihood of developing any disorder was higher in the heavy drinking category than those who did not meet criteria, 29.2% versus 17.3%.
Bryant et al. [26] 2014	Australia	To investigate the prevalence and predictors of psychological outcomes 3–4 years after a wildfire.	Cohort Study	Outcome measures included the abbreviated version of PCL-5, PHQ-9, K6 scale, and an abbreviated AUDIT.	Among the high impact group, 15.6% had probable wildfire related PTSD, 12.9% had probable MDD, and 24.7% had rates of heavy drinking.
Bryant et al. [40] 2017	Australia	To examine how separation from an attachment figure as a child during a trauma can influence long-term attachment styles and future psychopathology.	Comparative Cohort Study	Outcome measures included parental presence during the wildfire, Experiences in Close Relationships scale, and the PCL-5.	Children exposed to bushfires and children separated from their parents during that time had higher scores on the avoidant attachment scale as adults. An avoidant attachment style was positively associated with PTSD symptoms.
Bryant et al. [41] 2017	Australia	To examine how social connections between survivors of a disaster are linked to mental health outcomes.	Cohort Study	Outcome measures included social support questions, the PCL-5, and the PHQ-9.	Analyses found that identifying social support was associated with less depression, and being nominated by others as a support was associated with less PTSD.
Bryant et al. [42] 2018	Australia	To examine the changing prevalence and predictors of psychological outcomes in communities 5 years post-wildfire.	Cohort Study	Outcome measures included the abbreviated PCL-5, PHQ-9, the K6, and the AUDIT-C.	In the high-impact group, the rate of probable fire-related PTSD was 10.9%, general PTSD was 18.7%, MDE was 10.9%, and heavy alcohol use was 23.2%.
Byrne et al. [43] 2006	Australia	To determine the prevalence and risk factors for vicarious traumatization post-wildfire.	Cross-Sectional Study	Outcomes included IES, Vicarious Traumatization Questionnaire, and Coping Strategies Indicator.	Demographic factors were not found to be significant risk factors for vicarious traumatization.
Caamano-Isorna et al. [44] 2011	Spain	To analyze the impact of a wildfire on respiratory systems and mental health.	Cohort Study	Outcome of interest was the drug dispensing rates for anxiolytics/hypnotics and drugs for obstructive airway disease 12 months before and after the wildfires.	Male non-pensioners saw a relative increase of 12.2% and male pensioners of 15.88% in the defined daily doses for anxiolytics and hypnotics.
Camilleri et al. [45] 2010	Australia	To evaluate the impact of a wildfire on current health and wellbeing.	Cross-Sectional Study	Outcome measures included K10 and a survey on perceived wellbeing	A total of 39% reported their lives were more difficult after the bushfire. While, 19.5% were found to have high to very high rates of psychological distress.
Cherry and Haynes [14] 2017	Canada	To examine how a wildfire impacted mental and physical health.	Cohort Study	Outcome measures included the HADS.	It was reported that 16.7% of those evacuated had scores indicative of moderate to severe anxiety or depression
Clayer et al. [13] 1985	Australia	To evaluate immediate and long-term health effects 12 months after a wildfire.	Comparative Cross-Sectional Study	The outcome was evaluated on the GHQ.	Rates of alcoholism and drug problems approximately tripled pre-bushfire to post. Mental illness increased from 0.79% pre-bushfire to 3.01% post-bushfire.
Cowlishaw et al. [46] 2021	Australia	To examine rates of significant anger problems following a bushfire and how this relates to mental health.	Cross-Sectional Study	Outcome measures included the DAR-5, PCL-4, PHQ-9, and the K6.	Among those with anger problems, 58.6% had probable PTSD, 43.2% had probable depression, and 34.5% had K6 scores indicative of severe mental illness.
Felix et al. [47] 2015	United States	To study post-traumatic growth and coping mechanisms in parents and youth following a wildfire.	Cross-Sectional Study	Outcome measures included a scale to assess fire-related stress, the PTGI-SF, the MHI-5, the PFS, and the CERQ-Short Form.	Use of positive reappraisal coping techniques was associated with greater post-traumatic growth in youth and approached significance in parents.
Forbes et al. [48] 2015	Australia	To examine how anger and patient factors can impact mental health outcomes following a wildfire.	Cross-Sectional Study	Outcome measures included the PCL-5, the PHQ-9, the AUDIT, and the AAQ.	Analyses showed significant indirect effects for anger and stressful life events on mental health outcomes.
Gallagher et al. [49] 2019	Australia	To examine how voluntary group involvement can impact post-disaster mental health.	Cohort Study	Outcome measures included group involvement, the PCL-C, and the PHQ-9.	Curvilinear relationship between group involvement and PTSD at both times of follow-up but no significant relationship with depression.
Gallagher et al. [50] 2017	Australia	To examine how attachment styles influence depression and PTSD following a wildfire.	Cross-Sectional Study	Heterosexual couples were surveyed regarding their PTSD and depression symptoms and attachment styles.	Male partners with avoidant attachment styles were associated with depression and PTSD symptoms in both partners. Female partners with avoidant attachment styles were associated with more PTSD and depression symptoms in themselves.
Gallagher et al. [51] 2016	Australia	To evaluate if separation from family peri-wildfire disaster modifies the relationship between attachment style and mental health outcomes.	Cross-Sectional Study	Outcome measures included separation status, the ECR, the PHQ-9, and the PCL-5.	Separation from loved ones during the fire was associated with higher PTSD symptoms but not depression symptoms. There was an association between attachment anxiety and depression.
Hashoul-Andary et al. [52] 2016	Israel	To study the relationship between anxiety sensitivity, distress tolerance, and psychopathology post-trauma.	Cohort Study	Outcome measures included the ASI, DTS, PDS, the IDAS, and the Panic Attack Questionnaire-short form.	The 1-month levels of emotional avoidance and distress predicted the emotional distress intolerance and degree of distress at 3 months. The level of emotional distress intolerance at 3 months strongly predicted the degree of distress post-trauma at 6 months.
Hertz-Picciotto, I [53] Abstract 2020	United States	To study children and adolescents’ mental health 6 months post-wildfire.	Cross-Sectional Study	Outcome measures included changes in depressive mood, anxiety, and sleep symptoms.	A total of 21.7% of children and 28.7% of adolescents had changes in 3 or more behaviours or symptoms.
Ho et al. [55] 2014	Singapore	To study the acute physical and psychological symptoms during seasonal haze.	Cross-Sectional Study	Outcome measures included personal views on what is considered to be a dangerous PSI value, physical symptoms, and the IES-R.	Respondents who perceived lower PSI values as dangerous were more likely to be experiencing more physical symptoms or have higher IES-R scores.
Hooper et al. [54] 2018	Australia	To study how post-traumatic symptoms, coping strategies, and post-traumatic growth are related.	Cross-Sectional Study	Outcome measures included the IES-R, the Brief COPE Inventory, and the PTGI.	Greater post-traumatic symptoms and post-traumatic growth were associated with the utilization of avoidant, problem-focused, and emotion-focused coping strategies.
Jones et al. [16] 2002	United States	To study psychosocial functioning and short-term mental health consequences in children 6 weeks after a wildfire.	Cross-Sectional Study	Outcome measures were obtained from the DICA-R. Self-report measures included the IES, the STAI-C, and the FQ-C. Parents received the PTSD component of the DIS and the IES.	There was an average of 5 PTSD symptoms reported by children in the high loss (HL) group, and 4.2 in the low loss (LL) group. Participants of the HL group had an average score of 39.9 on the IES, and 23.4 in the LL group. There was no significant association between symptoms among parent and child pairs.
Jones et al. [56] 1994	United States	To examine short-term consequences of a wildfire on mental health in children and adolescents.	Comparative Cross-Sectional Study	Outcome measures were obtained from the DICA-R, the IES, and the FQ-C.	Victims met an average of 2.8 out of 5 PTSD criteria and control participants met an average of 1.6 criteria.
Jones et al. [78] 2003	United States	To examine the rates of PTSD, depression, and anxiety post-wildfire.	Comparative Cohort Study	The initial follow-up outcome measures included the PTSD module DIS, the IES, the BDI, FQ-A, and the STAI. The second follow-up outcome measures included the DIS, the IES, and the FQ-A.	At the first point of follow-up, the victim group had significantly higher scores for PTSD and depression. At the second point of follow-up, the victim group had a significant decline in total score and intrusive psychological symptoms on the IES.
Kirsch et al. [20] 2016	United States	To study longitudinal disaster recovery following a wildfire.	Cohort Study	Outcome measures were obtained from CASPER assessments designed for the study, and the mental health questions were based on the CDC BRFSS.	At initial point of follow-up, 54.8% of adults reported someone in the household experiencing depressed mood or hopelessness and sleeping problems. At the second point of follow-up, property damage resulted in a 19.3% greater likelihood of depression and/or hopelessness.
Marshall et al. [57] 2007	United States	To evaluate the prevalence of PTSD and MDD among individuals who had sought disaster relief from a wildfire 3 months prior.	Cross-Sectional Study	Outcome measures included the PCL-5 and the PHQ-9.	A total of 24% had probable PTSD and 33% had probable MDD.
Mcdermott and Palmer [19] 1999	Australia	To identify students experiencing significant emotional distress and depression after a wildfire and provide interventions.	Cross-Sectional Study	Outcome measures included the IES, the RMA, and the BDS.	A reported 12% of students were experiencing moderate-severe emotional distress symptoms, 4.7% had symptoms of depressive illness, and 14.1% had symptoms of high-trait anxiety.
McDermott et al. [58] 2005	Australia	To identify children and adolescents who require mental health support and interventions after experiencing a wildfire.	Cross-Sectional Study	Outcome measures included the PTSD-RI and the SDQ.	A reported 12.1% had moderate PTSD, and 9.0% had severe–very severe PTSD. There were significantly higher scores in the primary grades (4–6), then junior or senior grades (7–12).
Mcdermott and Palmer [59] 2002	Australia	To study depression and emotional distress in children and adolescents post-wildfire and association with developmental stage.	Cross-Sectional Study	Outcome measures included the IES, the RMA, and the BDS.	Multivariate analyses suggested that the IES score, the RMA score, grade at school, and experiencing evacuation significantly predicted the BDS index. BDS scores were lowest in middle grades. Adjusted emotional distress scores were lowest in the lower and high grades.
McFarlane et al. [18] 1987	Australia	To perform a longitudinal assessment of children, examining the evolution of psychological morbidity after a wildfire.	Comparative Cohort Study	Outcome measures obtained via parent and teacher administered Rutter Questionnaires.	From teacher questionnaires, the caseness rate at 2, 8, and 26 months was 1.8%, 6.5%, and 12%, respectively. There were variable results in comparison to the control group.
McFarlane and Van Hooff [23] 2009	Australia	To examine the rate of PTSD and other psychological disorders in adults who had experienced a wildfire during childhood.	Comparative Cohort Study	Outcome measures included the CDI, the AUDIT, and the IES-R.	A total of 36.7% of the bush-fire exposed participants and 31.7% of the controls met criteria for DSM-IV disorder in their lifetime. When DSM diagnoses were analyzed, the only one more significantly prevalent in the exposed group was lifetime history of specific phobia.
McFarlane et al. [60] 1997	Australia	To examine the prevalence of mental health problems following a bushfire.	Comparative Cohort Study	Outcome measures included the 28 item GHQ. The second part of the study involved a subset of victims. Outcome measures included the GHQ and the DIS.	From the first follow-up, the mean GHQ score was 5.6, and 42% met criteria as possible psychiatric cases. At the second point of follow-up, 23% of the surveyed victims met criteria for psychiatric caseness.
McFarlane [79] 1987	Australia	To examine post-traumatic symptoms and pathology in children following a wildfire.	Cohort Study	Outcome measures were obtained from Rutter Parent and Teacher Questionnaires.	The post-traumatic phenomena did not decrease in children from 8 to 26 months post-bushfire. There was significant correlation between the 2 month teacher symptom score and the 26 month scores. There was no 1:1 relationship between post-traumatic phenomena and psychological disorder.
Mellon et al. [61] 2009	Greece	To assess the relationship between an external locus of control and levels of psychopathology.	Comparative Cross-Sectional Study	Outcome measures were the Brown Locus of Control scale and the SCL-90-R.	Participants who resided in the wildfire impacted area had a higher external locus of control views and higher global levels of psychopathology (GSI). There was a statistically significant correlation between levels of external locus of control and psychopathology in those with greater trauma.
Molyneaux et al. [62] 2020	Australia	To study rates of interpersonal violence amongst communities impacted by a wildfire and the relationship with mental health.	Cross-Sectional Study	Outcome measures included participant experience of violence, the PCL-5, the PHQ-9, and the AUDIT-C.	There were more reports of experiencing violence in high-impact regions versus medium and low, 7.4% versus 0–1%. Experiencing violence predicted increased symptoms of depression and PTSD symptoms among women but not men.
Moore et al. [63] 2006	Canada	To examine if increases in particulate matter (PM2.5 and PM10) created by wildfires were linked to changes in physician visits.	Cohort Study	Outcome measures included PM2.5, PM10 levels and physician billing for visits related to respiratory cardiovascular and mental health disorders.	There was no significant difference in weekly rates for physician visits for respiratory or cardiovascular diseases or mental health disorders during the time of worsening air quality
Moosavi et al. [64] 2019	Canada	To assess the prevalence rates of likely PTSD, MDD, and GAD in patients of an after-hours family medicine clinic 18 months post-wildfire.	Cross-Sectional Study	Outcome measures included the PCL-5, the PHQ-9, and the GAD-7.	The rate for likely PTSD, MDD, and GAD was 13.6%, 24.8%, and 18.0%, respectively.
Papadatou et al. [65] 2012	Greece	To examine rates and risk factors for PTSD and depression in adolescents who had experienced a wildfire 6 months prior.	Cross-Sectional Study	Outcome measures included the WEQ, CRIES-13, the DSRS, and the Kidcope-Adolescent Version.	Depression and PTSD symptom scores differed between boys and girls. While, 29.4% of adolescents reported symptoms above the cutoff score for PTSD, and 20% for depression.
Papanikolaou et al. [66] 2011	Greece	To examine the psychological distress and morbidity following a wildfire.	Comparative Cross-Sectional Study	Outcome measures included the SCL-90-R.	The victim group had significantly higher symptom scores in somatization, depression, anxiety, hostility, phobic anxiety, obsession, and paranoia. There was a 43.6% case rate in the victim group, and 29.8% in the control group.
Papanikolaou et al. [67] 2011	Greece	To examine the psychological status and personal perceptions of a population impacted by a wildfire.	Comparative Cross-Sectional Study	Outcome measures included the SCL-90-R.	The fire exposure group had significantly higher scores on 7 out of 9 of the primary scales in the SCL-90-R, and on the Global Severity Index and Positive Symptom Total.
Parslow and Jorm [68] 2006	Australia	To determine the extent that tobacco use is impacted by a traumatic experience and symptoms of PTSD.	Cohort Study	Participants answered questions about wildfire experience, tobacco use, and completed the TSQ.	A reported 5% of participants screened positive for PTSD. In the regression analysis, increased levels of smoking were associated with a higher number of fire-related experiences but not PTSD symptoms.
Parslow et al. [69] 2006	Australia	To study risk factors for PTSD following a wildfire.	Cohort Study	Initial outcome measures included the GADS and the short form of the Eysenck Personality Questionnaire. At the second point of follow-up, participants also completed the TSQ.	A total of 5% of participants after the wildfire met criteria for PTSD. Overall, 6 variables were strongly associated with PTSD symptoms, female gender, fewer years of education, prior history of depression or anxiety, evacuation, injury or death of a loved one from the fire, and the degree of peritraumatic emotions.
Psarros et al. [70] 2017	Greece	To examine the relationship between insomnia and PTSD one month after a wildfire.	Cross-Sectional Study	Outcome measures included the SCL-90-R and the Athens Insomnia Scale.	A reported 46.7% met criteria for PTSD. Insomnia rates were higher in those with PTSD, at 79.1% versus 49% of those without.
Ritchie et al. [71] 2020	Canada	To study the prevalence of PTSD, GAD, and MDD in college students 18 months after experiencing a wildfire.	Cross-Sectional Study	Outcome measures included the PHQ-9, GAD-7, the PCL-5, the AUDIT, and the DUDIT.	The one month prevalence rate for likely MDD, GAD, and PTSD was 23.4%, 18.7%, and 11.0%, respectively. It was found that 15.5%, 13.5%, and 4.4% of participants screened positive for high risk drinking, problematic drug use, and moderate to high nicotine dependence.
Scher and Ellwanger [72] 2009	United States	To examine potential risk and protective factors for post-disaster adjustment and pathology.	Cohort Study	Two time-points of follow-up. Outcome measures included the FIQ, PTCI, BAI, BDI-II, and the PILL.	At the first point of follow-up, analyses found that female gender and negative fire-related cognitions were associated with increased anxiety symptoms. Increased negative fire-relation cognitions were associated with increased depression symptoms.
Silveira et al. [73] 2021	United States	To study mental health consequences and associated risk factors after a wildfire.	Comparative Cross-Sectional Study	Outcomes were assessed using the PCL-5, the PHQ-9, and the GAD-7.	PCL-5 scores were significantly higher for those directly exposed. PHQ-9 and GAD-7 scores were higher in those directly or indirectly exposed vs. not exposed.
Tally et al. [74] 2013	United States	To assess the impact of a wildfire on patients who utilized the public mental healthcare system.	Cross-Sectional Study	A regularly scheduled survey post-wildfire contained additional questions on the impact of the wildfires, evacuation status, and need for additional services.	A reported 18.1% of evacuees sought additional mental health services, compared to 8% of individuals who did not evacuate the area despite living in an evacuation zone, and 2.1% of those who lived in non-evacuation areas. Analyses revealed that the act of evacuation was significant for all 8 fire impact questions.
Verstraeten et al. [75] 2020	Canada	To assess the development of PTSD-like symptoms in perinatal women following exposure to a wildfire and protective factors.	Cross-Sectional Study	Outcome measures included the IES-R, the PDI, the PDEQ, the short-form SSQ, and the CD-RISC.	A total of 26% qualified for probable PTSD. Both peritraumatic distress and dissociative experience scores were positively associated with post-traumatic symptoms. Analyses found only social support satisfaction had a moderating effect on the relationship between peritraumatic distress (when it was not significantly high) and post-traumatic symptom scores.
Yelland et al. [76] 2010	Australia	To examine if wildfire disaster exposure variables or child demographics are associated with the severity of PTSD symptoms in youth.	Cross-Sectional Study	Outcome measures included the PTSD-RI-R and bushfire impact/experience questionnaire.	Average PTSD symptom score was 18.41; 17% had symptoms in the moderate range and 10% in the severe–very severe range. In analysis, relevant risk factors were perceived threat to life, ongoing loss or disruption in life, and younger age.
Zeller et al. [77] 2015	Israel	To explore the relationship between self-compassion and trauma-related psychopathology.	Cohort Study	Outcome measures included the SCS, the IDAS, the MAAS, and the Carmel Trauma Questionnaire.	Self-compassion did not have an impact on overall wellbeing post-wildfire. Higher levels of self-compassion were associated with lower levels of depression at all 3 time-points and anxiety at 3 and 6 months.

Abbreviations: Acronyms: SCL-90-R: The Symptom Checklist-90-Revised instrument; PST: Positive Symptom Total; GSI: Global Severity Index; WHOQOL-BREF: Abbreviated World Health Organization Quality of Life; MHI-5: Mental Health Inventory; GAD: Generalized Anxiety Disorder; GAD-7: Generalized Anxiety Disorder-7; AUDIT: Alcohol Use Disorder Identification Test; DUDIT: Drug Use Disorder Identification Test; MDD: Major Depressive Disorder; PHQ-9: Patient Health Questionnaire; PTSD: Post-Traumatic Stress Disorder; PCL-5: PTSD Checklist for DSM 5; PSQI-A: Pittsburgh Sleep Quality Index and its Addendum for PTSD; PTCI: Post-Traumatic Cognitions Inventory; WCQ: Ways of Coping Questionnaire; K6: Kessler Psychological Distress Scale-6; CD-RISC: the Connor Davidson Resilience Scale; CPSS: Child PTSD Symptom Scale; PHQ-A: The Patient Health Questionnaire Adolescent Version; HADS: Hospital Anxiety and Depression Scale; CRAFFT Questionnaire: Car, Relax, Alone, Forget, Friends, Trouble; RSE: Rosenberg Self-Esteem Scale; AUDIT-C: Alcohol Use Disorder Identification Test-Consumption; MDE: Major Depressive Episode; IES: Impact of Events Scale; K10: Kessler Psychological Distress Scale-10; GHQ: General Health Questionnaire; DAR-5: Dimensions of Anger Reactions Scale-5; PCL-4: the four-item version of the PTSD Checklist; PTGI-SF: Posttraumatic Growth Inventory-Short form; PFS: Protective Factor Survey; CERQ-Short Form: Cognitive Emotion Regulation Questionnaire; AAQ: Anger Attacks Questionnaire; ECR- Experiences in Close Relationships; PCL-C: the PTSD Checklist-Civilian Version; ASI: Anxiety Sensitivity Index, DTS: Distress Tolerance Scale; PDS: Post-Traumatic Diagnostic Scale; IDAS: Inventory of Depression and Anxiety Symptoms; PSI: Pounds per square inch; IES-R: Impact of Events Scale-Revised; COPE: Coping Orientations to Problems Experienced; PTGI: Posttraumatic Growth Inventory; DICA-R: Diagnostic Interview for Children and Adolescents-revised; FQ-C: Fire Questionnaire-Child Form; STAI-C: State-Trait Anxiety Inventory for Children; DIS: Diagnostic Interview Schedule; BDI: Beck Depression Inventory; FQ-A: Fire Questionnaire-Adult Form; STAI: State-Trait Anxiety Inventory; CASPER: Community Assessments for Public Health Emergency Response; CDC BRFSS: Centre for Disease Control and Prevention’s Behavioral Risk Factor Surveillance System; RMA: Revised Manifest Anxiety; BDS: Birleson Depression Inventory; PTSD-RI: Post-Traumatic Stress Disorder Reaction Index; SDQ: Strengths and Difficulties Questionnaire; CDI: Composite International Diagnostic Interview; PM2.5: particulate matter 2.5 microns; PM10: particulate matter 10 microns; WEQ: Wildfire Experience Questionnaire; CRIES-13: Children’s Revised Impact of Event Scale; DSRS: Depression Self-Rating Scale; TSQ: Trauma Screening Questionnaire; GADS: Goldberg Anxiety and Depression Scale; FIQ: Fire Impact Questionnaire; PTCI: Posttraumatic Cognitions Inventory; BAI: Beck Anxiety Inventory: BDI-II: Beck Depression Inventory-II; PILL: Pennebaker Inventory of Limbic Languidness; PDI: Perinatal Depression Inventory; PDEQ: Peritraumatic Dissociative Experiences Questionnaire; SSQ: Social Support Questionnaire; PTSD-RI-R: Post-traumatic Stress Disorder Reaction Index for Children-Revised; SCS: Self-Compassion Scale; MAAS: Mindful Attention Awareness Scale. #—Number.

## Data Availability

All data used in this review article are in public domain as described in the methods section.
